# A paternal methyl donor-rich diet altered cognitive and neural functions in offspring mice

**DOI:** 10.1038/mp.2017.53

**Published:** 2017-04-04

**Authors:** D P Ryan, K S Henzel, B L Pearson, M E Siwek, A Papazoglou, L Guo, K Paesler, M Yu, R Müller, K Xie, S Schröder, L Becker, L Garrett, S M Hölter, F Neff, I Rácz, B Rathkolb, J Rozman, G Ehninger, M Klingenspor, T Klopstock, E Wolf, W Wurst, A Zimmer, H Fuchs, V Gailus-Durner, M Hrabě de Angelis, K Sidiropoulou, M Weiergräber, Y Zhou, D Ehninger

**Affiliations:** 1 Molecular and Cellular Cognition Lab, German Center for Neurodegenerative Diseases (DZNE), Bonn, Germany; 2 Department of Neuropsychopharmacology, Federal Institute for Drugs and Medical Devices (BfArM), Bonn, Germany; 3 Department of Physiology, Medical College of Qingdao University, Qingdao, Shandong, China; 4 Department of Psychiatry and Psychotherapy, University of Cologne, Faculty of Medicine, Cologne, Germany; 5 German Mouse Clinic, Institute of Experimental Genetics, Helmholtz Zentrum München, German Research Center for Environmental Health, Neuherberg, Germany; 6 Friedrich-Baur-Institut, Department of Neurology, Ludwig-Maximilians-Universität München, Munich, Germany; 7 Institute of Developmental Genetics, Helmholtz Zentrum München, German Research Center for Environmental Health, Neuherberg, Germany; 8 Institute of Pathology, Helmholtz Zentrum München, German Research Center for Environmental Health, Neuherberg, Germany; 9 Institute of Molecular Psychiatry, Medical Faculty, University of Bonn, Bonn, Germany; 10 Chair of Molecular Animal Breeding and Biotechnology, Gene Center, Ludwig-Maximilians-Universität München, Munich, Germany; 11 Member of German Center for Diabetes Research (DZD), München-Neuherberg, Germany; 12 Department of Internal Medicine I, University Hospital Carl Gustav Carus, Technical University Dresden, Dresden, Germany; 13 Molecular Nutritional Medicine, Else Kröner-Fresenius Center, Technische Universität München, Freising-Weihenstephan, Germany; 14 German Center for Vertigo and Balance Disorders, University Hospital Munich, Campus Grosshadern, Munich, Germany; 15 DZNE, German Center for Neurodegenerative Diseases, Munich, Germany; 16 Munich Cluster for Systems Neurology (SyNergy), Adolf-Butenandt-Institut, Ludwig-Maximilians-Universität München, Munich, Germany; 17 Chair of Developmental Genetics, Technische Universität München, c/o Helmholtz Zentrum München, German Research Center for Environmental Health, Neuherberg, Germany; 18 Chair of Experimental Genetics, Center of Life and Food Sciences Weihenstephan, Technische Universität München, Freising-Weihenstephan, Germany; 19 Department of Biology, University of Crete, Vassilika Vouton, Heraklio, Greece

## Abstract

Dietary intake of methyl donors, such as folic acid and methionine, shows considerable intra-individual variation in human populations. While it is recognized that maternal departures from the optimum of dietary methyl donor intake can increase the risk for mental health issues and neurological disorders in offspring, it has not been explored whether paternal dietary methyl donor intake influences behavioral and cognitive functions in the next generation. Here, we report that elevated paternal dietary methyl donor intake in a mouse model, transiently applied prior to mating, resulted in offspring animals (methyl donor-rich diet (MD) F1 mice) with deficits in hippocampus-dependent learning and memory, impaired hippocampal synaptic plasticity and reduced hippocampal theta oscillations. Gene expression analyses revealed altered expression of the methionine adenosyltransferase *Mat2a* and BK channel subunit *Kcnmb2*, which was associated with changes in *Kcnmb2* promoter methylation in MD F1 mice. Hippocampal overexpression of *Kcnmb2* in MD F1 mice ameliorated altered spatial learning and memory, supporting a role of this BK channel subunit in the MD F1 behavioral phenotype. Behavioral and gene expression changes did not extend into the F2 offspring generation. Together, our data indicate that paternal dietary factors influence cognitive and neural functions in the offspring generation.

## Introduction

Epigenetic regulation of gene expression is centrally facilitated by the addition of methyl groups (CH_3_) to DNA cytosine nucleotides.^1^ Abundant methylation in gene promoters, particularly in CpG residues, recruits chromatin modifiers and transcriptional repressors in a manner that usually silences gene expression.^2, 3^ Such methylation-based control of gene expression is thought to have an important role in numerous physiological processes including the regulation of neural functions and behavior.^4, 5^

DNA methylation patterns are sensitive to diet. Food-derived nutrients such as choline and methionine are prominent sources of methyl donors.^6^ Dietary folate is critical to the synthesis of S-adenosylmethionine (SAM), another important methyl substrate.^7^ Insufficient maternal folic acid (FA) intake has long been associated with elevated risk for neural tube defects^8^ (reviewed by Blom *et al.*^9^). Dietary intake of methyl donors is subject to substantial variability within human populations.^6, 10^ Variation in methyl donor consumption may derive from seasonal food availability, which is sufficient to alter genome-wide CpG methylation patterns.^11, 12^ Countries like the United States have fortification programs that enforce FA supplementation, while other countries do not.^13^ On a population level, such fortification programs led to an increase in serum folate concentrations of up to 150% or greater.^14, 15^ In addition to FA fortification, methyl donors such as vitamin B12 are present in vitamin supplements but are also added to diverse foods including energy drinks.^16^

Despite the well-documented positive effects of methyl donor supplementation, there is increasing evidence that excessive consumption of methyl donors may have deleterious effects and result in adverse health outcomes in directly exposed individuals.^17^ Disruption of fetal development and fetal resorption were observed when pregnant mice were fed a FA-rich diet.^18, 19^ In humans, maternal consumption of high doses of FA during pregnancy may elevate the risk for asthma, atopic dermatitis and allergies in their children.^20, 21, 22^ Exposing females to a folic acid-rich diet during a period before mating caused reversal learning deficits in offspring mice (Henzel *et al.*, unpublished data), suggesting that exposure of female germ cells to excess FA may be sufficient to result in behavioral alterations in the next generation.

Whether increased paternal methyl donor intake has effects on offspring health has not been addressed to date. Given the emerging appreciation that sperm contribute epigenetic information to offspring,^23^ we performed a first study to test the hypothesis that a methyl donor-enriched paternal diet^24^ (enriched for FA, L-methionine, choline, zinc, betaine and vitamine B_12_), administered for 6 weeks before mating, alters behavioral, metabolic and neurophysiological functions in offspring.

## Materials and methods

### Mice

All mice were housed under SPF conditions with 2–5 mice per cage on a 12:12 h light/dark cycle with food and water available *ad libitum*. Mouse studies were approved by ‘Landesamt für Natur, Umwelt und Verbraucherschutz Nordrhein-Westfalen’ (Recklinghausen, Germany), ‘Regierung von Oberbayern’ (Munich, Germany) (in accordance with the German Animal Health and Welfare Act, German Federal Law, §8 Abs. 1 TierSchG) or by the Chancellor’s Animal Research Committee at Qingdao University, China (in accordance with National Institutes of Health guidelines). Young adult C57BL/6J males were purchased from Charles River (Sulzfeld, Germany). After an acclimation phase of several weeks, the mice were randomly assigned to two groups. The control diet (CD) group was fed a standard Teklad global 18% protein rodent-breeding diet (T.2918.12, Envigo, Indianapolis, IN, USA) while the methyl donor-rich diet (MD) group received a specialized 3MS ZM diet based on the same formula (T.110835, Envigo). As previously described^24^ the 3MS ZM diet was supplemented with the following (per 1 kg chow): 7.5 g L-methionine, 15 g choline, 15 g betaine, 15 mg FA, 1.5 mg vitamin B_12_ and 150 mg zinc. After 6 weeks of feeding, males were individually mated with young adult 129S6/SvEv female mice purchased from Taconic (Ejby, Denmark) to generate the F1 offspring of MD and CD fathers. We chose to perform experiments in F1 offspring animals on a C57BL/6 J × 129S6/SvEv background because these mice are isogenic and they are often better learners than the inbred parental mouse strains,^25, 26^ which is consistent with the general notion of hybrid vigor associated with outbreeding.^27^ F2 offspring were created by mating male F1 offspring of MD or CD fathers with female F1 offspring of CD fathers. Contact between males and offspring was excluded and offspring were left undisturbed with their dam until weaning at the age of 21 days. Adult CD and MD offspring (F1, F2) were assessed in balanced sex ratios. All experiments were conducted blind to group assignment.

### Morris water maze

We assessed spatial learning and memory using a hidden version of the Morris water maze as previously described (see also Supplementary Methods, for further details).^28, 29^

### Context fear conditioning

To assess conditioned fear, we used a video fear conditioning system (Med Associates, Fairfax, VT, USA) essentially as previously described.^30^ Mice received a single training session of 181 s with one 0.75 mA shock (1 s) after 120 s. During testing, one day later, animals were returned to the conditioning chambers for a 3 min context test and the conditioned fear responses (% time freezing) were quantified using an automated system (Med Associates).

### Accelerating rotarod

An accelerating rotarod (Bioseb, Vitrolles, France) was used to measure motor coordination, balance and motor learning abilities.^31^ Mice were placed on the rotarod and the rod rotations were subsequently accelerated from 4 to 40 r.p.m. during the 5 min trial period. Trials were terminated when animals fell off the rod, showed passive cycling or when 5 min had elapsed, whichever came first. Animals were given three trials with inter-trial intervals of 15 min. Average data from the three trials are presented.

### Startle reflex and pre-pulse inhibition

Startle reflex and pre-pulse inhibition were assessed using a startle apparatus setup (Med Associates) including four identical sound-attenuating cubicles. Background noise was 65 dB, and startle pulses were bursts of white noise (40 ms). A session was initiated with a 5-min acclimation period followed by five presentations of leader startle pulses (110 dB) that were excluded from statistical analysis. Trial types for the pre-pulse inhibition included four different pre-pulse intensities (67, 69, 73 and 81 dB); each pre-pulse preceded the startle pulse (110 dB) by a 50 ms inter-stimulus interval. Each trial type was presented 10 times in random order, organized in 10 blocks, each trial type occurring once per block. Inter-trial intervals varied from 20 to 30 s.

### Open field

The test apparatus (ActiMot, TSE, Bad Homburg, Germany) was a transparent and infrared light-permeable acrylic test box (internal dimensions: 45.5 cm × 45.5 cm × 39.5 cm) equipped with two pairs of light-beam strips. After being undisturbed for 30 min before testing, animals were allowed to freely explore the test arena for 20 min. Illumination levels were set at ~150 lux in the corners and 200 lux in the middle of the test arena. We compared total distance traveled, number of rearings and total time spent in the center during the 20 min period across groups.

### Grip strength

We used a grip strength meter system (Bioseb) to determine grip strength of the mice. The task exploits the tendency of the mice to grasp a horizontal metal grid while being pulled at the tail. During the setup for the trial, the mice grasped a special adjustable grid mounted on a force sensor. Mice were then allowed to hold on to the grid with either two or four paws. Each mouse was given three trials over the course of 1 min. Mean values were used to represent the grip strength of a mouse.

### Hot plate

The mice were placed on a metal surface maintained at 52±0.2 °C (Hot plate system, TSE). Locomotion on the hot plate was constrained by a circular acrylic glass wall (20 cm high, 18 cm in diameter). Mice remained on the plate until they performed one of three behaviors regarded as indication of a painful sensation: hindpaw licking, hindpaw shake/flutter or jumping. Forepaw licking and lifting were ignored because these behaviors represent components of normal grooming. The response latency was recorded to the nearest 0.1 s. To avoid tissue injury, a 30 s cutoff time was used.

### *In vitro* electrophysiology

After killing mice with isoflurane, we extracted brains and generated slice preparations in ice-cold artificial cerebrospinal fluid saturated with 95% O_2_ and 5% CO_2_ and containing (in mM): 120 NaCl, 20 NaHCO_3_, 3.5 KCl, 2.5 CaCl_2_, 1.3 MgSO_4_ 1.25 NaH_2_PO_4_ and 10 d-glucose. We generated 400 μm sagittal slices with a Leica VT1200S (Wetzlar, Germany) vibratome and allowed slices to recover at 23 °C for at least 60 min. Recordings were performed in a submerged chamber continuously perfused with oxygenated artificial cerebrospinal fluid (29 °C) at a rate of 3 ml min^−1^. For extracellular recordings of field excitatory postsynaptic potentials, we used platinum-iridium electrodes in CA1 stratum radiatum. Schaffer collaterals were stimulated with bipolar platinum electrodes (pulse duration: 100 ms). For the input–output function, we stimulated slices with incrementally increasing current (from 10 to 100 mA in 10 mA increments) and recorded field excitatory postsynaptic potential responses. We induced long-term potentiation (LTP) with high-frequency stimulation (1 s, 100 Hz). We determined initial field excitatory postsynaptic potential slopes and normalized them to the average baseline field excitatory postsynaptic potential slopes.

### *In vivo* electrophysiology

The implantation procedure, recordings and data analyses were performed as described previously (see also Supplementary Methods, for further details).^32, 33^

### Virus microinjection into CA1 of the dorsal hippocampus

High titers of AAV virus (>1 × 10^13^ GC /ml, from Vector Biolabs, Malvern, PA, USA) engineered to overexpress Kcnmb2 (AAV1-hSyn1-Kcnmb2-IRES-GFP) or control virus (AAV1-hSyn1-GFP) were stereotaxically injected into the CA1 region of the dorsal hippocampus through a 30-gauge Hamilton microsyringe at four sites (0.3 ul per site) at the following coordinates (mm) relative to bregma: AP −2, ML ±1, DV −1.4; or AP -3, ML ±2.6, DV −1.5). The viral injection speed was 0.1 μl min^−1^. After infusion, the microsyringe was left in place for an additional 10 min to ensure optimal virus diffusion in the tissue. After surgery, mice were treated with antibiotics daily for 2 weeks and their health was monitored every day. The viral infection in CA1 region was confirmed by assessment of GFP fluorescence on coronal brain slices (250 μm in thickness) freshly cut with a vibratome (Leica), and the images were acquired using an Olympus (Tokyo, Japan) FluoView 1000 Laser Scanning Confocal Microscope.

### Microarray-based gene expression analyses

We performed gene expression microarray analyses in collaboration with a specialized service facility (DNA Microarray Facility, University Medical Center, Göttingen, Germany) using the ‘Low RNA Input linear Amplification Kit Plus, One Color’ protocol (Agilent Technologies, Santa Clara, CA, USA, 2007; Cat. No.: 5188–5339) and the Agilent ‘One Color RNA Spike-In Kit’ (Agilent Technologies, 2007; Cat. No.: 5188-5282) following the manufacturer’s standard protocol. Global gene expression analysis was applied using the Mouse GE 4x44K v2 Microarray Kit (Cat. No. G4846A). 600 ng of total RNA were used as starting material to prepare cDNA. cDNA synthesis and *in vitro* transcription were performed according to the manufacturer’s recommendation. Quantity and efficiency of the labeled amplified cRNA were assessed using a NanoDrop (Thermo Scientific, Wilmington, DE, USA) ND-1000 UV-VIS Spectrophotometer version 3.2.1. The hybridizations were performed for 17 h at 10 r.p.m. and 65 °C in a hybridization oven (Agilent). Washing and staining of the arrays were done according to the manufacturer’s recommendation. Cy3 intensities were detected by one-color scanning using an Agilent DNA microarray scanner (G2505B) at 5 μm resolution. Scanned image files were inspected for artifacts and then analyzed.

Intensity data were extracted using Agilent’s Feature Extraction (FE) software (version 9.5.3.1) including a quality control based on internal controls using Agilent’s protocol GE1_107_Sep09. All chips passed the quality control and were analyzed using the Limma package of Bioconductor. The microarray data analysis consists of the following steps: (1) between-array normalization; (2) global clustering and PCA-analysis; (3) fitting the data to a linear model; (4) detection of differential gene expression; and (5) over-representation analysis of differentially expressed genes. We applied quantile-normalization to the log_2_-transformed intensity values as a method for between-array normalization. Microarray data have been deposited to GEO under accession number GSE90504.

### qPCR

Total RNA was reverse transcribed into cDNA using the iScript cDNA Synthesis Kit (Bio-Rad, Hercules, CA, USA). qPCR was performed using a StepOnePlus Real-Time PCR system and commercially available TaqMan Gene Expression Assays (Applied Biosystems, Foster City, CA, USA). *Actb* was used as housekeeping control for all samples and expression of the candidate genes was analyzed relative to that observed in the respective control group.

### Pyrosequencing

Targeted DNA methylation analysis, based on pyrosequencing, was performed in collaboration with a specialized service company (Varionostic, Ulm, Germany). For pyrosequencing analysis, samples were prepared according to standard procedures using a Vacuum Prep Tool. 40–50 μl PCR product was immobilized to 3 μl Streptavidin Sepharose HP beads (GE Healthcare, Chicago, IL, USA) followed by annealing to 2 μl sequencing primer (5 μM) for 2 min at 80 °C. For more information on primer sequences and assays used see Supplementary Tables 7 and 8. Analyses of CpGs were performed using Pyro Q-CpG software (Biotage, Uppsala, Sweden). Bisulfite conversion with subsequent pyrosequencing did not distinguish methylated and hydroxymethylated cytosines (5 mC vs 5 hmC, respectively).^34^

### MeDIP-chip

MeDIP-chip analysis was performed on sperm and hippocampus samples at a specialized service company (ImaGenes, Berlin, Germany) according to standardized protocols. In brief, genomic DNA was extracted and quality was controlled on an agarose-gel. DNA was fragmented using a Bioruptor (Diagenode, Liège, Belgium), which was followed by another quality control step using a Bioanalyzer (Agilent Technologies). Next, methylated DNA was enriched using a MethylMiner enrichment kit (Invitrogen, Carlsbad, CA, USA) enriching 5 mC (but not 5 hmC).^34^ After quality control via qPCR, whole-genomic DNA amplification was performed using a WGA kit (Sigma Aldrich, St Louis, MO, USA) followed by a second qPCR for quality control. DNA methylation analyses were performed on mouse 3 × 720 K CpG Island Plus RefSeq Promoter Arrays (NimbleGen, Madison, WI, USA). Enriched and input samples were labeled with Cy5/Cy3 and dye incorporation rates were controlled with a NanoDrop (Thermo Scientific). After co-hybridization, the arrays were washed and scanned, followed by quality control of the images. Peaks were called using the NimbleGen NimbleScan (v2.3) software by identifying peak positions from signal ratio data (IP vs input) in chromosomal bins of 500 bp yielding an average of peak numbers per bin in accordance with the manufacturer’s guidelines. Differentially methylated regions were defined as chromosomal loci, where across either the three treatment samples or control samples there were two or more peaks, whereas the corresponding regions in the other sample contained none. Ingenuity pathway analyses (IPA, Qiagen, Venlo, The Netherlands) focused on gene sets associated with nearby candidate regions for differential methylation peaks (<5 kb upstream of TSS). MeDIP-chip data have been deposited to GEO under accession number GSE90504.

### Indirect calorimetry

Metabolic assessments were performed at room temperature (23 °C) with a 12:12 h light/dark cycle in the room. Each mouse was placed individually in the metabolic cage for a period of 21 h. Wood shavings and paper tissue were provided as bedding material. Metabolic cages were setup in a ventilated cabinet continuously supplied with fresh air. High precision CO_2_ and O_2_ sensors measured the difference in CO_2_ and O_2_ concentrations in air volumes flowing through individual cages. Combined with parallel airflow measurements, this enabled calculation of oxygen consumption (expressed as ml O_2_ per hour per animal) over a given time period. The system also monitored CO_2_ production, and, hence, it was possible to determine the respiratory exchange ratio (RER) and heat production. The RER corresponds to the ratio VCO_2_/VO_2_. Delta RER values were calculated for each animal by subtracting the minimal RER value from the maximal RER value of the respective animal.

### Clinical chemistry

Blood samples were taken from isoflurane-anesthetized mice by puncturing the retro-bulbar sinus with non-heparinized glass capillaries (1.0 mm in diameter; Neolab, Heidelberg, Germany). Blood samples were collected in heparinized tubes (Li-heparin, KABE, Nümbrecht, Germany). Each tube was immediately inverted five times to achieve a homogeneous distribution of the anticoagulant. Li-heparin-coated tubes were stored at room temperature for 1 to 2 hours. Afterwards, cells and plasma were separated by a centrifugation step (10 min, 5000 *g*, 8 °C). Plasma samples were then transferred into an Eppendorf tube and diluted 1:2 with distilled water. The solution was mixed for a few seconds to prevent clotting and was then centrifuged again (10 min, 5000 *g*, 8 °C). Lactate and urea measurements were performed using a Beckman-Coulter AU 480 autoanalyzer and reagents from Beckman-Coulter (Brea, CA, USA).

### Metabolomics

Liver tissue samples were sent to a specialized company (Chenomx, Edmonton, AB, Canada) for 1D nuclear magnetic resonance (NMR) data acquisition and targeted profiling. After sample preparation NMR spectra were acquired on a Varian two-channel VNMRS 600 MHz NMR spectrometer equipped with a HX 5 mm probe. The pulse sequence used was a 1D-tnnoesy with a 990 ms presaturation on water and a 4 s acquisition time. Spectra were collected with 32 transients and four steady-state scans at 298 K. Spectra were processed using the Processor module in Chenomx NMR Suite 7.6. Compounds were identified and quantified using the Profiler module in Chenomx NMR Suite 7.6.

### Statistics

Statistical comparisons were performed using GraphPad Prism (v7) (La Jolla, CA, USA). Details of statistical tests used and sample sizes are provided in the main text, figure legends and within tables. Unless otherwise noted, parametric tests were performed with no assumptions violated for the particular statistical test.

## Results

### Offspring of methyl donor supplemented fathers displayed cognitive impairments

**Figure 1:**
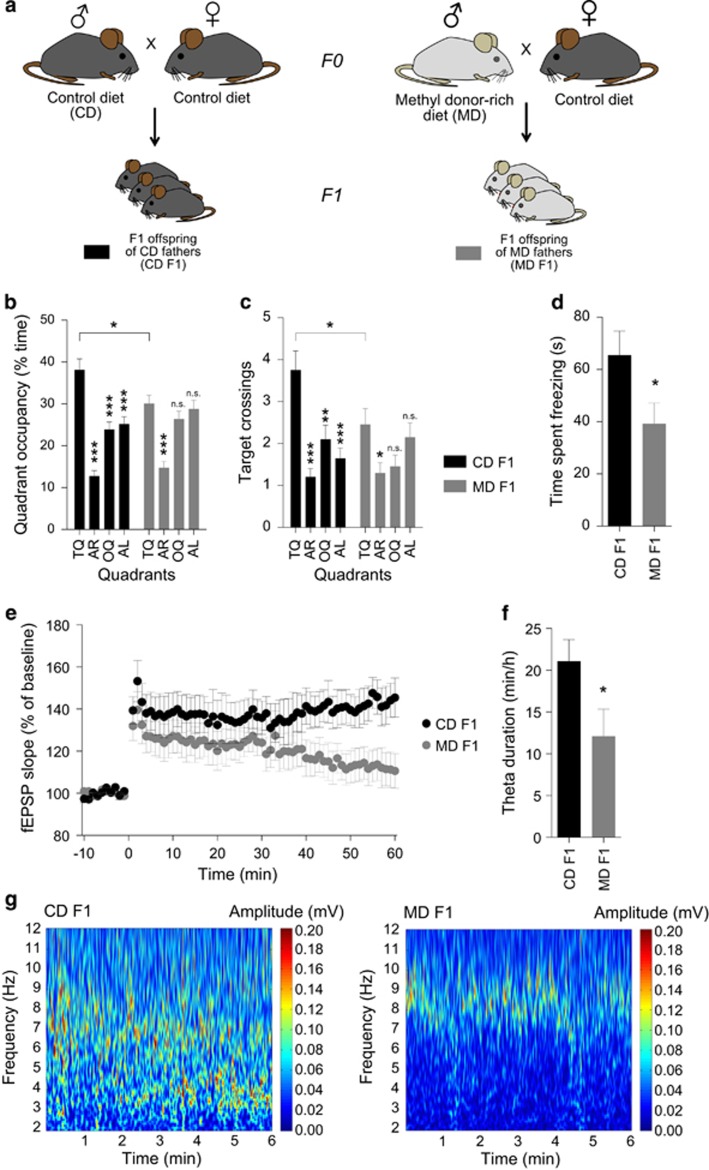
Impaired hippocampus-dependent learning and memory and altered hippocampal functions in MD F1 offspring mice. (**a**) Experimental design. (**b** and **c**) Quadrant occupancy measures (**b**) and target crossings (**c**) during a probe trial given after completion of water maze training (*n*=20 mice per group). Pool quadrants: TQ; AR; OQ; and AL. (**d**) Freezing during a context test given 1 day after training in a contextual fear conditioning paradigm (CD F1: *n*=17 mice; MD F1: *n*=14 mice). (**e**) Measurements of long-term potentiation at Schaffer Collateral/CA1 synapses in acute brain slices (CD F1, *n*=7 mice; MD F1, *n*=8 mice). (**f**) Quantification of oscillatory activity in the theta range measured during video-EEG analysis of CD F1 and MD F1 mice (*n*=11 mice per group). (**g**) Representative time frequency plots show reduced typical theta segments in a MD F1 mouse relative to a CD F1 mouse. All graphs display mean ± s.e.m. *P<0.05, **P<0.01, ***P<0.001. AL, adjacent left; AR, adjacent right; CD, control diet; EEG, electroencephalogram; MD, methyl donor-rich diet; OQ, opposite quadrant; TQ, target quadrant.

We tested spatial learning and memory in a hippocampus-dependent, hidden version of the Morris water maze in the adult F1 offspring of fathers that either received the MD or standard CD (*n*=20 mice per group). In this task, animals were trained to find an escape platform hidden underneath the water surface in a constant location of a pool filled with opaque water. To assess how accurately animals had learned the position of the escape platform, we gave a single probe trial after completion of training. The probe trial data revealed altered swim patterns in MD F1 mice that are indicative of spatial learning impairments in these animals (Figure 1): There was a significant interaction between paternal diet (between-subjects factor) and quadrant (within-subjects factor) with regards to quadrant occupancy (two-way analysis of variance (ANOVA), *P*=0.0317) and target crossing (*P*=0.0381) measures, demonstrating differences in probe trial swim patterns between groups. Dunnett’s multiple comparisons test revealed that CD F1 mice showed significantly higher occupancy, as well as target crossing values in the target quadrant than in all the other quadrants (Figure 1), indicating highly targeted searching of the mice, while this was not the case in MD F1 mice (Figure 1). Further analyses demonstrated significant group differences with regards to target quadrant occupancy (unpaired *t*-test, *P*=0.0194) and target crossings (unpaired *t*-test, *P*=0.0365) with overall higher values in CD F1 mice than MD F1 mice (for additional probe trial measures as well as training trial data, see Supplementary Table 1). We did not observe group differences in the hippocampus-independent, visible version of the Morris water maze (for data and results of statistical analyses, see Supplementary Table 1). Altogether, these data indicate that motivational, perceptual or motor differences were unlikely to account for the water maze phenotype of MD F1 offspring. Moreover, additional behavioral assessment revealed normal exploratory behavior, anxiety-related behaviors, motor skills and sensory processing in the F1 offspring of MD fathers (Supplementary Table 1). These results indicate that spatial learning in a hidden version of the Morris water maze was impaired in the F1 offspring of MD fathers relative to controls.

We also examined hippocampus-dependent learning and memory in CD F1 and MD F1 mice using an associative contextual fear-conditioning paradigm (CD F1, *n*=17 mice; MD F1, *n*=14 mice). In this task, mice were allowed to explore a novel environment (that is, the conditioning chamber) that was subsequently paired with a mild foot shock delivered through the metal grid floor of the chamber. We measured the strength of associative learning by placing the animals back into the conditioning chamber 1 day post training and measured the time the animals spent freezing during a 3 min test. We did not find group differences with regards to baseline activity during the training day (that is, during the 2 min period before the onset of the shock; data not shown), indicating that overall locomotor activity was normal in MD F1 mice. We also found no group differences with regards to shock reactivity (data not shown), suggesting that MD F1 mice showed no obvious aberrations in nociceptive perception. The context test, 1 day after training, revealed reduced freezing levels in MD F1 mice compared with the controls (Figure 1; unpaired *t*-test, *P*=0.0441), demonstrating impaired contextual fear conditioning in the offspring of fathers on the MD. Together, these analyses revealed impairments in two hippocampus-dependent learning and memory tasks in MD F1 mice relative to CD F1 controls.

### Impaired hippocampal long-term potentiation in offspring of methyl donor supplemented fathers

Activity-dependent synaptic modifications in the hippocampus are thought to represent a cellular correlate of learning and memory processes that depend on this brain structure.^35^ To determine if hippocampal synaptic plasticity was altered in the offspring of fathers that received the MD, we measured LTP at Schaffer Collateral/CA1 synapses in acute brain slices prepared from offspring derived from MD or CD fathers (CD F1, *n*=7 mice; MD F1, *n*=8 mice). These experiments revealed reduced levels of LTP in the offspring of MD fathers relative to controls (Figure 1; two-way ANOVA with the between-subjects factor paternal diet and the within-subjects factor time: effect of paternal diet, *P*=0.1177; effect of time, *P*<0.0001; paternal diet × time interaction, *P*<0.0001; *t*-test, last 10 min of recordings, *P*=0.0205). In contrast, basal synaptic transmission was normal in MD offspring slices (data not shown), indicating that some aspects of synaptic function were unaffected in MD offspring.

### Altered theta oscillations in offspring of methyl donor supplemented fathers

Hippocampal theta oscillations are thought to be important in hippocampal information processing underlying learning.^36^ We, therefore, employed deep, intrahippocampal CA1 electroencephalogram recordings to measure local field potentials in awake and freely behaving F1 offspring of CD and MD fathers (long-term video-electroencephalogram) (*n*=11 mice per group). Analyses of oscillatory activity in the theta range revealed reduced overall theta activity in the F1 offspring of fathers on the MD relative to controls (Figure 1; *t*-test, *P*=0.0432). Theta oscillations were reduced in MD offspring both during motor episodes (type I theta), as well as during behavioral inactivity (type II theta) (Supplementary Table 2). There was no measurable difference in motor activity between groups (Supplementary Table 2).

### Hippocampal gene expression analyses identified differentially expressed genes in MD F1 mice

Together, these results are consistent with the idea that epigenetic changes, induced in the paternal germ line by the MD, may be transmitted to the subsequent generation, where they influence behavioral and cognitive traits, as well as putative neurophysiological substrates thereof. We further examined this hypothesis by evaluating gene expression in the hippocampus of the offspring derived from MD or CD fathers (*n*=6 arrays per group; for each array we used pools of *n*=4 mice). Microarray analyses identified a set of differentially expressed genes in the F1 hippocampus (Supplementary Table 3). Notably, one of the genes downregulated in MD F1 hippocampus was *Mat2a* (Methionine Adenosyltransferase II alpha), the expression of which is known to be downregulated in response to L-methionine or SAM (that is, a component supplemented in the MD diet and a metabolite thereof).^37, 38, 39^ These analyses also revealed a reduced expression of *Kcnmb2*, which encodes for the BK channel beta subunit 2 and is a known regulator of neuronal excitability, synaptic plasticity and memory.^40^ Reduced *Mat2a* and *Kcnmb2* expression were confirmed by qPCR in an independent set of samples (Supplementary Table 4).

### Computational assessment of synaptic and network consequences of altered *Kcnmb2* expression

The BK channel beta subunit 2, encoded by *Kcnmb2*, has a role in the inactivation of BK currents.^41^ To explore a possible role of altered BK current inactivation in the electrophysiological phenomena changed in MD F1 offspring (hippocampal LTP, theta oscillations) we assessed the effects of decreasing BK channel inactivation on synaptic properties and integration *in silico* in a compartmental model of a CA1 pyramidal neuron, which has been validated extensively in the context of previous studies.^42, 43^ The results of these analyses are described in detail in Supplementary Results, as well as Supplementary Figures 1 and 2. In brief, the analyses indicate that decreasing BK channel inactivation resulted in impaired spatial integration of synaptic inputs in the model, suggesting that reduced *Kcnmb2* expression could contribute, in principle, to alterations in synaptic plasticity, theta oscillations and behavior observed in MD F1 mice.

### Hippocampal Kcnmb2 overexpression improved water maze impairments in MD F1 mice

**Figure 2:**
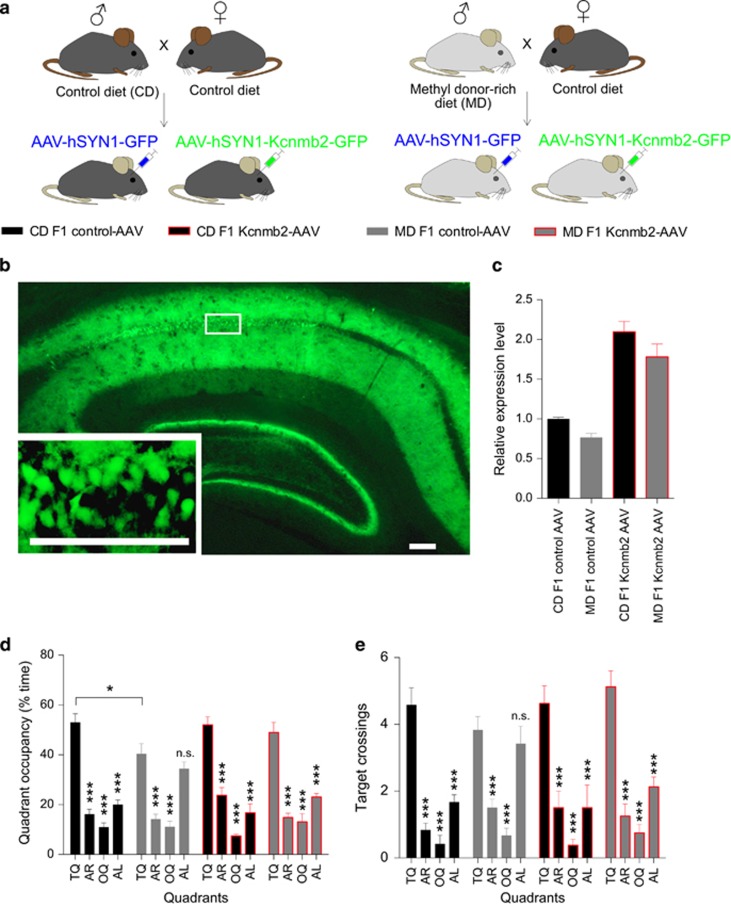
Hippocampal Kcnmb2 overexpression improved water maze impairments in MD F1 mice. (**a**) Experimental design. (**b**) Representative image of hippocampal slices infected with AAV1-hSyn1-Kcnmb2-IRES-GFP or control AAV1-hSyn1-GFP virus. Green, GFP, imaged 4 weeks after virus injection. Scale bar, 100 μm. (**c**) Relative *Kcnmb2* mRNA expression in the hippocampus after viral infection as determined by qPCR (*n*=5 mice per group; two-way ANOVA with the between-subjects factors paternal diet and AAV treatment: effect of paternal diet, *P*=0.0214; effect of AAV treatment, *P*<0.0001; paternal diet × AAV treatment interaction, *P*=0.7084). (**d** and **e**) Quadrant occupancy measures (**d**) and target crossings (**e**) during a probe trial given after completion of water maze training (CD F1 control-AAV, *n*=12 mice; MD F1 control-AAV, *n*=12 mice; CD F1 Kcnmb2-AAV, *n*=8 mice; MD F1 Kcnmb2-AAV, *n*=8 mice). Pool quadrants: TQ; AR; OQ; and AL. All graphs display mean±s.e.m. *P<0.05, **P<0.01, ***P<0.001. AL, adjacent left; ANOVA, analysis of variance; AR, adjacent right; CD, control diet; GFP, green fluorescent protein; MD, methyl donor-rich diet; OQ, opposite quadrant; TQ, target quadrant.

To further examine if *Kcnmb2* loss of function contributes to behavioral alterations in MD F1 mice, we addressed whether AAV-mediated overexpression of Kcnmb2 in hippocampus modified MD F1-related learning and memory impairments in the Morris water maze. Toward this end, high titers of AAV virus (>1 × 10^13^ GC /ml) engineered to overexpress Kcnmb2 (AAV1-hSyn1-Kcnmb2-IRES-GFP; Kcnmb2-AAV) or control virus (AAV1-hSyn1-GFP; control-AAV) were stereotaxically injected into the CA1 region of the dorsal hippocampus of CD F1 mice, as well as MD F1 mice at 4 weeks before training in a hidden version of the Morris water maze (Figure 2 and Supplementary Table 5). We gave a probe trial after completion of training to assess how accurately animals had learned the position of the escape platform. We confirmed the finding that the swim pattern during the probe trial was influenced by paternal diet and found that this was modulated by Kcnmb2 overexpression: Three-way ANOVA with the between-subjects factors paternal diet and AAV treatment and the within-subjects factor quadrant showed a significant quadrant x paternal diet interaction, as well as a significant quadrant x AAV treatment interaction for the quadrant occupancy measures (quadrant x paternal diet interaction, *P*<0.0001; quadrant × AAV treatment interaction, *P*=0.0178. Target crossings: quadrant × paternal diet interaction, *P*=0.1171; quadrant × AAV treatment interaction, *P*=0.0963). Dunnett’s multiple comparisons test revealed that CD F1 control-AAV mice, CD F1 Kcnmb2-AAV mice and MD F1 Kcnmb2-AAV mice showed significantly higher occupancy values in the target quadrant than in all the other quadrants, indicating highly targeted searching of the mice in these groups, while this was not the case in MD F1 control-AAV mice (Figure 2). The data indicate that hippocampal Kcnmb2 overexpression ameliorated the water maze phenotype of MD F1 mice, supporting that reduced *Kcnmb2* expression contributes to MD F1-related behavioral alterations.

### Targeted and genome-wide DNA methylation analyses identified differential methylation in MD F1 offspring

**Figure 3:**
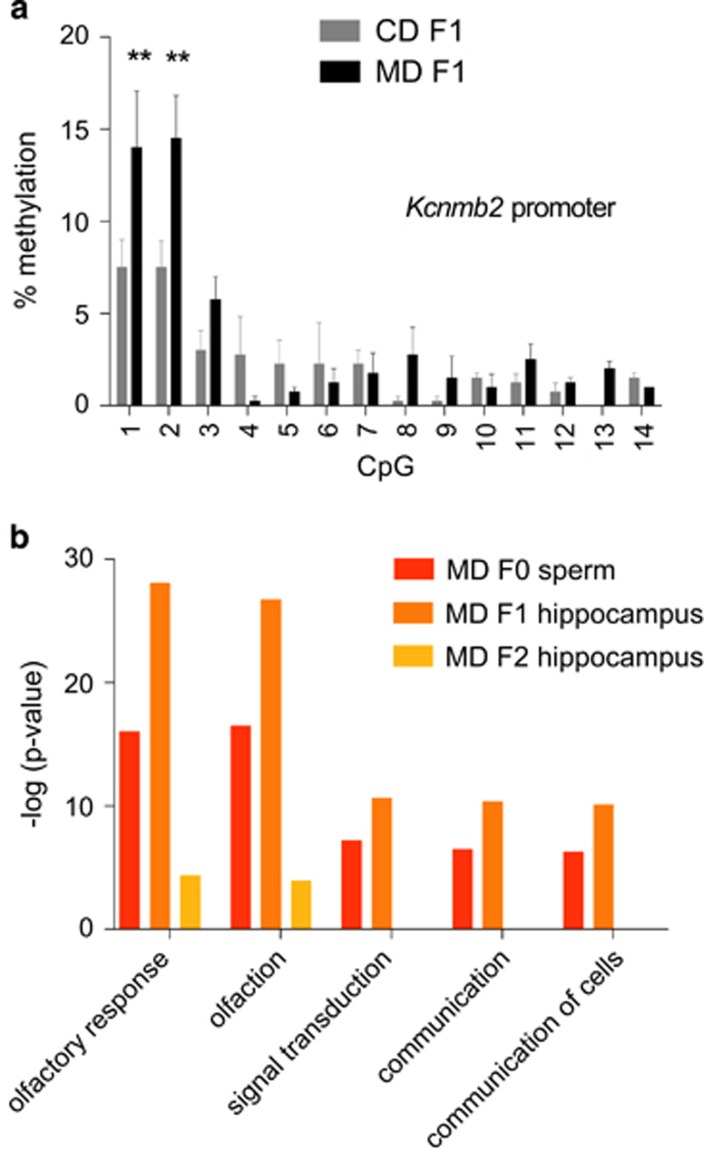
DNA methylation changes in hippocampal tissue of MD F1 mice. (**a**) Targeted bisulfite sequencing-based analysis of CpG methylation around the *Kcnmb2* transcription start site in hippocampal tissue of MD F1 and CD F1 mice (*n*=4 mice per group). (**b**) Genome-wide DNA methylation analyses using MeDIP-chip identified processes and pathways enriched among genes linked to hypermethylated regions in MD mice (fathers, F1 offspring, F2 offspring; for full results of the Ingenuity pathway analyses, see Supplementary Data Files 3). CD, control diet; MD, methyl donor-rich diet.

We next explored the possibility that altered DNA methylation in corresponding regulatory regions accounted for the differential gene expression in MD F1 offspring described above. We performed targeted bisulfite pyrosequencing to determine if CpG methylation around the transcription start site (TSS) of *Kcnmb2* was altered in the offspring hippocampus as a consequence of the paternal diet (*n*=4 mice per group). We found altered CpG methylation in MD F1 mice (Figure 3; two-way ANOVA with paternal diet as between-subjects factor and CpG position as within-subjects factor: effect of paternal diet, *P*=0.0896; effect of CpG position, *P*<0.0001; paternal diet x CpG interaction, *P*=0.0026), indicating that differential methylation of *Kcnmb2* regulatory regions could underlie altered *Kcnmb2* expression in MD F1 mice.

In order to gain insights into possible DNA methylation changes associated with the MD diet on a more global scale, we performed MeDIP-chip analyses of sperm of MD and CD fathers (*n*=3 arrays per group; for each array we used pools of *n*=4 mice), as well as MD F1 and CD F1 offspring mice (*n*=3 arrays per group; for each array we used pools of *n*=4 mice). These studies provided a number of candidate regions with differential methylation peaks in the F0 and the F1 generation (Supplementary Data Files 1 and 2, respectively). We performed IPA comparing the gene set linked to these regions against the full set of genes. These analyses revealed a significant enrichment of processes related to olfaction among the gene set linked to hypermethylated regions in both MD F0 sperm and hippocampal tissue of MD F1 offspring (Figure 3; for full IPAs of genes linked to MD-hypermethylated regions, see Supplementary Data Files 3 and 4; for IPAs of genes linked to MD-hypomethylated regions, see Supplementary Data Files 5 and 6), reflecting the fact that many MD-hypermethylated regions were situated in the vicinity of olfactory receptor gene clusters.

### Limited evidence for transgenerational effects on gene expression and behavior in the grandpaternal offspring of methyl supplemented sires

**Figure 4:**
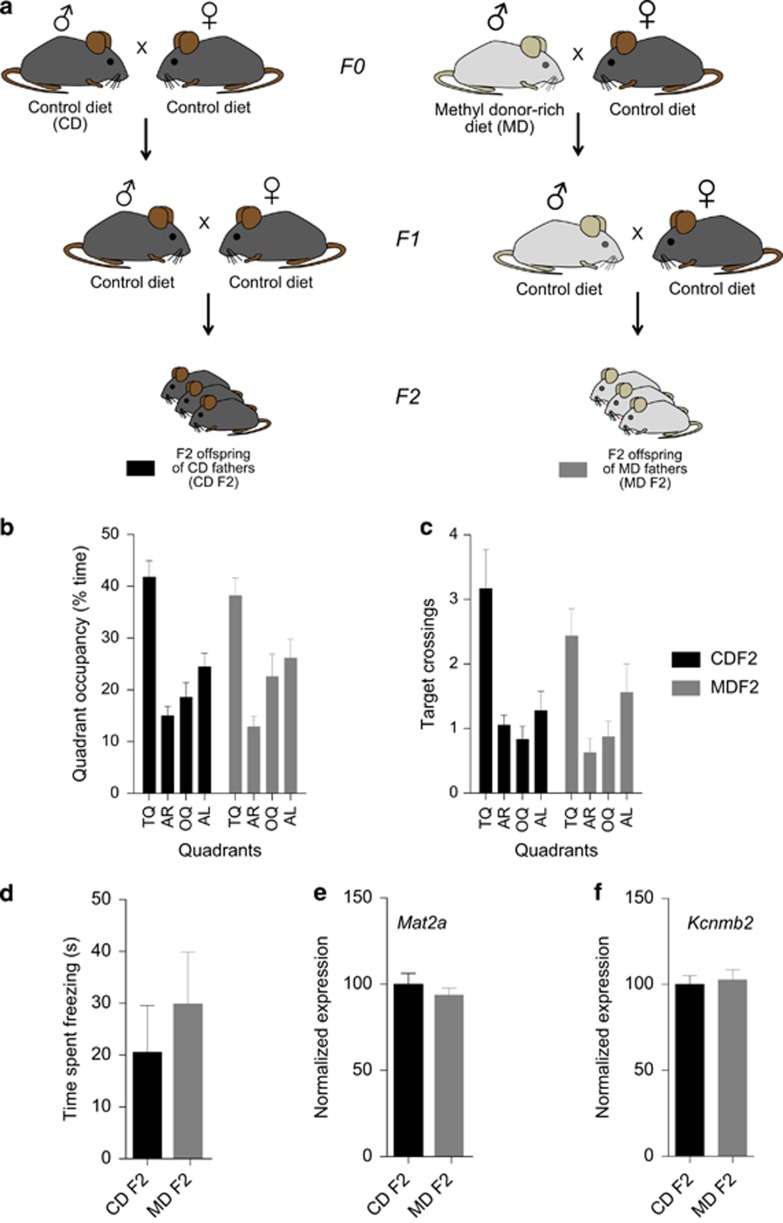
Grandpaternal exposure to a MD did not measurably affect learning and memory and hippocampal gene expression in F2 offspring mice. (**a**) Breeding scheme: male F1 offspring of MD and CD fathers were mated with CD F1 females to generate MD F2 and CD F2 offspring. (**b** and **c**) A probe trial performed after completion of training in a hidden version of the water maze revealed no obvious differences between MD F2 and CD F2 mice (CD F2, *n*=18 mice; MD F2, *n*=16 mice; (**b**), quadrant occupancy: two-way ANOVA with the between-subjects factor grandpaternal diet and the within-subjects factor quadrant: effect of grandpaternal diet, *P*=0.9141; effect of quadrant, *P*<0.0001; paternal diet × quadrant interaction, *P*=0.6842; (**c**), target crossings: two-way ANOVA with the between-subjects factor grandpaternal diet and the within-subjects factor quadrant: effect of grandpaternal diet, *P*=0.4356; effect of quadrant, *P*<0.0001; paternal diet × quadrant interaction, *P*=0.4648). Pool quadrants: TQ; AR; OQ; and AL. (**d**) MD F2 mice did not show measurably different freezing levels compared with CD F2 controls on a context test given 1 day after training in a contextual fear-conditioning task (CD F2, *n*=8 mice; MD F2, *n*=9 mice; *t*-test, *P*=0.5019). (**e** and **f**) qPCR-based quantification of hippocampal *Mat2a* and *Kcnmb2* expression did not reveal measurable differences between MD F2 and CD F2 mice (*n*=12 mice per group; *Mat2a*: *t*-test, *P*=0.4044; *Kcnmb2*: *t*-test, *P*=0.7334). ANOVA, analysis of variance; AL, adjacent left; AR, adjacent right; CD, control diet; MD, methyl donor-rich diet; qPCR, quantitative PCR; OQ, opposite quadrant; TQ, target quadrant.

We next asked if these methyl donor-rich-diet-induced behavioral and gene expression alterations persist beyond the F1 offspring generation. We generated F2 offspring of males exposed to either the MD or the control diet prior to mating. Behavioral assessment of the resulting F2 progeny revealed no clear group differences in the Morris water maze and a contextual fear-conditioning task (for details, see Figure 4, Supplementary Table 6). Microarray-based gene expression analyses of F2 hippocampal tissue failed to identify group differences (*n*=6 arrays per group; for each array we used pools of *n*=4 mice; data not shown). Furthermore, qPCR-based assessment of *Mat2a* and *Kcnmb2* expression also indicated no measurable effect of a grandpaternal MD on the expression of these genes (Figure 4). Comparison of genes linked to hypermethylated regions in a MeDIP-chip analysis of MD F2 vs CD F2 hippocampus, however, revealed a significant over-representation of olfactory receptor genes in MD F2 offspring hippocampus (Figure 3; see Supplementary Data File 7 for MD/CD F2 MeDIP-chip data set; for full IPA of genes linked to MD F2-hypermethylated regions, see Supplementary Data File 8; for IPAs of genes linked to MD F2-hypomethylated regions, see Supplementary Data File 9), similar to the findings in MD F0 sperm and MD F1 hippocampus, suggesting that MD-associated DNA methylation changes may partially extend to the grandpaternal offspring of fathers that transiently received a MD prior to mating.

### Paternal methyl diet elicited metabolic changes in F1 offspring animals

We next examined the possible influence of paternal methyl donor diet supplementation on offspring metabolic parameters. Liver-based metabolomic assessments in MD/CD F0 and MD/CD F1 mice (*n*=5 mice per group in both experiments) identified candidate metabolites with differential abundance in MD F0 and/or MD F1 liver (for details, see Supplementary Figure. 3). Measurements of two selected metabolites altered in MD F1 liver (that is, lactate, urea) showed similar changes in plasma in an independent cohort of animals (Supplementary Figure. 3; CD F1: *n*=22 mice; MD F1: *n*=23 mice). Histopathological analyses of liver samples suggested reduced glycogen content in MD F1 livers and the presence of focal steatotic changes in MD F1 mice (Supplementary Figure. 3). Indirect calorimetry revealed reduced variability of RER in MD F1 mice, suggesting a bias toward lipid oxidation in these mice (Supplementary Figure. 3). Together, these data suggest paternal dietary effects on energy metabolism in MD F1 mice and they indicate that physiological changes in MD F1 mice are not only restricted to the brain but also affect peripheral tissues.

## Discussion

In the present study, we examined whether a transient exposure (for 6 weeks) of male mice to a MD before mating exerts intergenerational effects on learning and behavior in offspring mice. We found impairments in the F1 offspring of MD fathers in two hippocampus-dependent learning and memory tasks (Morris water maze and contextual fear conditioning), as well as alterations in hippocampal synaptic plasticity and oscillations in the theta range, that is, cellular and network processes thought to be important for memory-related hippocampal information processing. These behavioral and neurophysiological changes in MD F1 mice were associated with altered hippocampal gene expression, including the expression of *Kcnmb2*—encoding a BK channel subunit—and *Mat2a*—encoding a methionine adenosyltransferase, as well as promoter methylation changes around the TSS of *Kcnmb2*. The F2 offspring of MD fathers, in contrast, did not show obvious behavioral differences to controls and displayed normal *Mat2a* and *Kcnmb2* expression, suggesting that the paternal exposure to the MD affected the subsequent generation but left F2 offspring unaffected (but see below).

Decreased *Mat2a* expression could reflect a compensatory response to elevated availability of methyl substrates. Mat1a and Mat2a levels in cultured liver cells are sensitive to SAM^39^ and L-methionine^38^ availability signifying that Mat2a regulation could respond to dietary methyl levels. A compensatory reduction of *Mat2a* expression could function to reduce SAM biosynthesis^44^ under conditions of enhanced methyl donor availability. *MAT1A* polymorphisms and plasma methionine levels interact in a manner that predicts circulating SAM levels in healthy humans^45^ supporting that Mat1 and Mat2 function is central in regulating SAM levels under physiological conditions, *in vivo*.

Metabolic assessments suggested possible intergenerational effects of the MD diet on energy metabolism in offspring mice; a consistent finding reproduced across liver and plasma was a reduction of lactate concentrations, possibly indicative of reduced levels of anaerobic glycolysis in these mice. Effects on carbohydrate metabolism were also suggested by reduced maltose levels in liver (maltose is an intermediate metabolite of glycogen metabolism) and were also supported by the qualitative observation of reduced liver glycogen content in MD F1 offspring during histological examination. Histological assessment of liver also revealed focal steatosis in MD F1 mice, but not CD F1 animals. Indirect calorimetry showed reduced maximal RER values in MD F1 offspring, indicating a potential bias toward lipid oxidation^46^ in these mice. These results complement a growing literature demonstrating inheritance of altered metabolic traits as a function of paternal diet in laboratory rodents.^47, 48, 49, 50^

Recently, paternal deficiency in dietary folate was shown to induce anatomical birth defects in mouse offspring.^51^ However, little is known about paternal derived intergenerational effects of dietary methyl donors and brain function. High-dose gestational folate exposure in rats caused altered seizure threshold *in vivo*^52^ demonstrating that neuronal and synaptic physiology is affected by methyl donor excess through maternal exposure. In the current study, we found cognitive alterations and impaired LTP in MD F1 mice indicative of neurological, and particularly, synaptic impairments of a paternal origin. In MD F1 mice, the BK channel gene *Kcnmb2* showed reduced expression and elevated promoter methylation. BK channels are large conductance Ca^2+^-activated K^+^ channels involved in regulating membrane excitability and action potential termination via its rapid hyperpolarization response to Ca^2+^ influx (reviewed by Lee and Cui^40^). Genetic deletion of *Kcnmb2* in chromaffin cells elicited delayed action potential repolarization and reduced action potential firing under current injection.^53^ *Kcnmb2* expression is bidirectionally modulated by pharmacological activation and inactivation.^54^ Thus, it is plausible that altered BK function could lead to synaptic plasticity impairments. Our computational approach supported that decreasing BK channel inactivation is sufficient to impair spatial summation of synaptic inputs.

It is possible that dietary influences on BK channel subunit expression and function could be modulated by polyamine abundance. Polyamines such as spermine and spermidine are synthesized from decarboxylated SAM.^55^ Thus, methyl donor intake has the potential to influence polyamine levels in the periphery^56^ and the brain^57^ and since polyamine levels influence BK channel activity^58^ it is conceivable that high methyl donor diets exert influences via polyamine synthesis. In our study, MD fathers showed elevated hepatic trimethylamine levels (another poly/biogenic amine) which has been shown to be elevated upon choline administration,^59^ supporting the notion that disruptions to biogenic amine abundance could have altered BK channel function. Further experiments are warranted to elucidate the intergenerational influences of paternal diets on polyamine biosynthetic pathways.

Methylation arrays identified additional differentially methylated regions in olfactory receptor gene clusters in MD F1 mice. This raises the possibility that increased bioavailability of methyl substrates drives persistent hypermethylation in AT-rich, heavily methylated loci associated with this most abundant vertebrate gene class.^60^ Future research can employ high-resolution bisulfite sequencing approaches to further define this phenomenon in conditions of excess methyl donor consumption in males.

Although significant enrichment of olfactory receptors among differentially methylated regions persisted into the F2 generation, the hippocampal dependent cognitive impairments did not. However, due to the breeding scheme, the F1 offspring animals used in the present study were isogenic while the F2 mice were not. Genetic heterogeneity in this intercross could have masked the detection of phenotypic differences in F2 offspring mice. Key future experiments could evaluate transgenerational influences of grandpaternal dietary methyl supplementation in isogenic lines. Likewise, performing the dietary manipulation in outcrossed mouse lines with subsequent determination of the intergenerational influences on behavior and physiology would help distinguish whether paternal methyl influences are masked under conditions of genetic heterogeneity. At present, additional research is necessary to identify the limits of paternal dietary influence on DNA methylation and other traits across generations, particularly in light of epidemiological evidence that male grandparental dietary abundance was associated with F2 health.^61, 62, 63^

Here we have demonstrated intergenerational effects on cognition, behavior and neurophysiological measures in response to a paternal MD. Since growing numbers of foods^64^ in certain countries are being fortified with methyl donors such as FA, increased comprehension of dietary methyl donor influences is warranted to prevent neurocognitive and metabolic alterations in subsequent generations. Our data indicate that an epidemiological assessment of paternal methyl donor consumption and the corresponding effects on offspring outcomes are justified in order to weigh potential costs and benefits of supplementation with FA and other methyl donors. Taken together, our study identified adverse effects of a paternal MD on cognitive and neural functions in offspring animals, raising the possibility that paternal dietary factors may be relevant causal factors for mental health issues in the subsequent generation.
