# Supplementary material for: A paternal methyl donor-rich diet altered cognitive and neural functions in offspring mice
Source: Mol Psychiatry. 2017 Apr 4;23(5):1345–55. doi: 10.1038/mp.2017.53 (PMC5984088; doi:10.1038/mp.2017.53)
Supplement: Supplementary Information [file mp201753x1.docx]

**Supplementary Information**

**Supplementary Methods**

**Morris water maze:** After handling mice to habituate them to experimental manipulations (2 min per animal for 7 days), mice were trained over two trials per day for 7 consecutive days to locate an escape platform hidden 1 cm underneath the water surface in a constant location of a pool (diameter: 120 cm). Training trials were terminated when the animal reached the escape platform or when 60 s had elapsed. To assess how accurately animals had learned the position of the escape platform, we conducted a probe trial after completion of training, during which the platform was removed from the pool and the search pattern of the animal was recorded by an automated system (Ethovision XT). With respect to the training trials, we analyzed escape latencies, thigmotaxis and distance travelled; regarding the probe trial, quadrant occupancy and target crossings were recorded. Additionally, we compared swimming speed, thigmotaxis, and cumulative distance to the platform during the probe trial across groups. To ensure that visually guided behavior was comparable between groups we completed the water maze experiments by delivering 4 visible training trials, during which the platform was marked by a clearly visible cue and had alternating spatial positions within the pool.

***In vivo* electrophysiology:** Positioning of the differential intracerebral electrode ((-)-lead) of the TA10ETAF20 Transmitter (DSI) targeting the hippocampal CA1 region was as follows: bregma -2 mm, lateral of bregma 1.5 mm (right hemisphere), dorsoventral (depth) 1.5 mm. The epidural reference electrode ((+)-lead) was placed on the cerebellar cortex at: bregma -6 mm, lateral of bregma 1 mm (right hemisphere). For postoperative pain management animals were administered carprofen (5 mg/kg sc., Rimadyl, Parke-Davis/Pfizer). Animals were allowed to recover for 10 days prior to subsequent recordings. Validation of EEG electrode placement was carried out post mortem.^1^ Ten days after radiotransmitter implantation, simultaneous video-EEG recordings from the hippocampal CA1 region were performed for 48 h using Dataquest ART 4.2 software (DSI) at a sampling rate of 1000 Hz with no a priori filter cut-off.

Complex EEG analysis was performed for spontaneous 48 h recordings at a sampling rate of 1000 Hz. Data segments with a length of 60 min each were extracted from the 48 h total recordings. Data segments were analyzed using complex Morlet wavelets to calculate both frequency and amplitude of oscillations as described previously.^1, 2^ The complex Morlet wavelet was used to analyze EEG data in the frequency range of 0.2 – 12 Hz with a step size of 0.1 Hz. A task-adjusted theta-oscillation detection criterion^2^ was applied to elaborate the frequency architecture of theta activity. Theta-positive oscillatory segments of 2.5 s duration^3^ were defined as follows (A_max_: maximum amplitude):

$$\frac{A_{\text{max(4-12Hz)}}\left[ mV \right]}{A_{\text{max(2-3.9Hz)}}\left[ mV \right]}>2$$

EEG segments identiﬁed as theta oscillation epochs were compared across groups. Furthermore, behavioral activity data were used to assign CA1 theta activity measurements to either active or non-active motor behavioral states. All EEG calculations were done using custom-made programs in Matlab (The MathWorks Inc., Version R2012b).

**Neuron computational model**: The compartmental model of a CA1 pyramidal was implemented in the NEURON simulation environment.^4^ The biophysical model of the CA1 pyramidal neuron has been described earlier.^5, 6^ The model consists of 183 compartments and includes a variety of passive and active membrane mechanisms known to be present in CA1 pyramidal cells. We assumed a uniform membrane resistance of Rm = 40 kΩ.cm^2^; a uniform intracellular resistivity Ra = 70 Ω.cm; and a specific membrane capacitance of 1.0 µF.cm^-2^. The resting membrane potential of the model neuron was set at -66 mV. Active mechanisms included, Hodgkin-Huxley-type Na^+^ currents (axonal: I^a^na; dendritic: I^d^na), voltage-dependent K^+^ currents (IKdr; IA; IM), a fast Ca^++^ and voltage-dependent K^+^ current, IfAHP; a slow Ca^++^-dependent K^+^ current, IsAHP; a hyperpolarization-activated non-specific cation current (Ih); a low-voltage activated calcium current IcaT; a persistent sodium current INap; and four types of Ca^++^- and voltage-dependent calcium currents (IcaN; IcaR; IcaL-1.3; IcaL-1.2). Channel equations, distributions and densities of Ina, IKdr and IA are described in more detail elsewhere.^5^ In order to model decreased BK channel inactivation, the mod file for I(AHP) was changed accordingly. AMPA and NMDA currents were activated on the apical dendrites of the CA1 neuron model depending on the experiment. For all experimental simulations, 10 different runs were conducted and, in each run, synapses on different dendritic segments were activated. In order to study the frequencies generated by the CA1 neuron model, we stimulated the neuron model with 15 synapses at 10 different dendritic segments. In order to maintain the output levels of excitability the same, we stimulated the CA1 neuron model with 100 synapses at 10 different dendritic segments. Power spectra were generated on the summed synaptic currents (AMPA, NMDA, and GABA_A_) generated by the pyramidal neurons in the network, averaged for 10 trials, over a 1 s period of steady-state persistent activity, 3 s after the end of the stimulus. The averaged synaptic currents were first decimated and then the mean square power spectrum was calculated using the periodogram method.

***Description of the fast calcium dependent potassium current*** $E_{K_{fAHP}}$:

$$I_{\left[K\right]}=g_{K}*O*(V-E_{Ca})$$

The channel has three states according to ^7^: active (O), non active (C) and inactivated (I).

The transition from C🡪O is calcium dependent. The transition from O🡪I is voltage dependent and is responsible for the presence of the fast afterhyperpolarization potential. The transition from I🡪C is voltage dependent and slow as well.

$$\begin{matrix} C\leftrightarrow O (K_{3}, K_{4}) \\ O\leftrightarrow I (K_{1}, 0) \\ I\leftrightarrow C (K_{2}, 0) \\ C+O+I=1 \end{matrix}$$

where K_1_, K_2_, K_4_ are described by function $\alpha_{2}(V)$and K_3_ by function $\alpha_{1}(V)$ and correspond to the transition rates between open (O), close (C) and inactivated (I) states:

$\alpha1(V)=\frac{1}{t_{\min}+\frac{1}{\frac{1}{t_{\max}-t_{\min}}+e((V-V_{half})/K)\cdot1ms^{-1}}}*1e8*([Ca]i)e2$ (114)

$\alpha_{2}(V)=\frac{1}{t_{\min}+e^{-(\frac{V-V_{half}}{K})\cdot1ms}}$ (115)

where

|  | $t_{\min}$ (ms) | $t_{\max}$ (ms) | $V_{half}$ (mV) | K (mV) |
| --- | --- | --- | --- | --- |
| K1 | 0.1 | - | -10 | 1.0 |
| K2 | 0.1 | - | -120 | -10.0 |
| K3 | 0.001 | 1.0 | -20 | 7.0 |
| K4 | 0.01 | - | -44 | -5.0 |

In order to simulate the decrease in the current inactivation, the following parameters were used:

|  | $t_{\min}$ (ms) | $t_{\max}$ (ms) | $V_{half}$ (mV) | K (mV) |
| --- | --- | --- | --- | --- |
| K1 | 0.1 | - | -10 | 1.0 |
| K2 | 0.1 | - | -120 | -70.0 |
| K3 | 0.001 | 1.0 | -20 | 50.0 |
| K4 | 0.01 | - | -44 | -5.0 |

**Supplementary Results**

We used a computation neuron model to study possible functional effects of decreased BK current inactivation. BK-mediated current underlies the fast afterhyperpolarization.^8^ Under physiological conditions, this current inactivates within a spike train.^7^ In addition, it is also found in the dendrites of CA1 pyramidal neurons^9^ where it could contribute to synaptic integration.^10^

We used a compartmental neuron model of a CA1 pyramidal cell, which has been validated extensively previously.^5, 6^ We modeled decreased BK channel inactivation by changing specific parameters in the mod file that simulates the current through the BK channels. As a result, the inactivation of the BK current was decreased when the model neuron was stimulated with current injection at the soma for 500 ms (**Supplementary Figure 1**). This effect resulted in slightly decreased neuronal excitability, since fewer action potentials were generated with the same amplitude of current injection (**Supplementary Figure 1**).

Next, we wanted to determine the effect of decreased BK channel inactivation on the synaptic properties and synaptic integration along the apical dendrites of the CA1 neuron model. We found that the decreased BK current inactivation did not affect the excitatory postsynaptic potential (EPSP) generated by stimulation of a single synapse (**Supplementary Figure 2**). However, when more synapses at different dendritic segments were stimulated, the resulting somatic EPSP was significantly decreased (**Supplementary Figure 2**), suggesting that spatial integration of the synaptic inputs was modified and resulted in decreased excitability at the soma. Furthermore, if a synapse was stimulated 5 times at 20 Hz frequency, we found that the 5th EPSP at the soma was significantly reduced when the BK current inactivation was decreased (**Supplementary Figure 2**). Finally, when the CA1 neuron received synaptic stimulation at 10 different dendritic segments at 20 Hz frequency, both the first and the fifth EPSP were significantly reduced, suggesting an overall reduction in excitability at the soma (**Supplementary Figure 2**). Next, we investigated the output frequency of the CA1 neuron model upon receiving incoming synaptic stimulation in the theta range. We found that, when the BK current inactivation was reduced, similar incoming stimulation resulted in significantly reduced power in the theta range (**Supplementary Figure 2**). However, at the same time, the neuronal excitability of the CA1 neuron model was also decreased compared to the control CA1 model. Therefore, we increased the incoming stimulation in order for the excitability of both the control and ‘decreased BK current inactivation’ models to be equal. Under this condition, the theta power was still reduced in the ‘decreased BK current inactivation’ model (**Supplementary Figure 2**).

**Supplementary References**

1. Siwek ME, Muller R, Henseler C, Trog A, Lundt A, Wormuth C *et al.* Altered theta oscillations and aberrant cortical excitatory activity in the 5XFAD model of Alzheimer's disease. *Neural Plast* 2015; **2015:** 781731.

2. Muller R, Struck H, Ho MS, Brockhaus-Dumke A, Klosterkotter J, Broich K *et al.* Atropine-sensitive hippocampal theta oscillations are mediated by Cav2.3 R-type Ca(2)(+) channels. *Neuroscience* 2012; **205:** 125-139.

3. Goutagny R, Krantic S. Hippocampal oscillatory activity in Alzheimer's disease: toward the identification of early biomarkers? *Aging Dis* 2013; **4**(3)**:** 134-140.

4. Hines ML, Carnevale NT. The NEURON simulation environment. *Neural Comput* 1997; **9**(6)**:** 1179-1209.

5. Poirazi P, Brannon T, Mel BW. Arithmetic of subthreshold synaptic summation in a model CA1 pyramidal cell. *Neuron* 2003; **37**(6)**:** 977-987.

6. Liebmann L, Karst H, Sidiropoulou K, van Gemert N, Meijer OC, Poirazi P *et al.* Differential effects of corticosterone on the slow afterhyperpolarization in the basolateral amygdala and CA1 region: possible role of calcium channel subunits. *J Neurophysiol* 2008; **99**(2)**:** 958-968.

7. Shao LR, Halvorsrud R, Borg-Graham L, Storm JF. The role of BK-type Ca2+-dependent K+ channels in spike broadening during repetitive firing in rat hippocampal pyramidal cells. *J Physiol* 1999; **521 Pt 1:** 135-146.

8. Adams PR, Constanti A, Brown DA, Clark RB. Intracellular Ca2+ activates a fast voltage-sensitive K+ current in vertebrate sympathetic neurones. *Nature* 1982; **296**(5859)**:** 746-749.

9. Sailer CA, Kaufmann WA, Kogler M, Chen L, Sausbier U, Ottersen OP *et al.* Immunolocalization of BK channels in hippocampal pyramidal neurons. *Eur J Neurosci* 2006; **24**(2)**:** 442-454.

10. Storm JF. Temporal integration by a slowly inactivating K+ current in hippocampal neurons. *Nature* 1988; **336**(6197)**:** 379-381.

**Supplementary Figures**

**
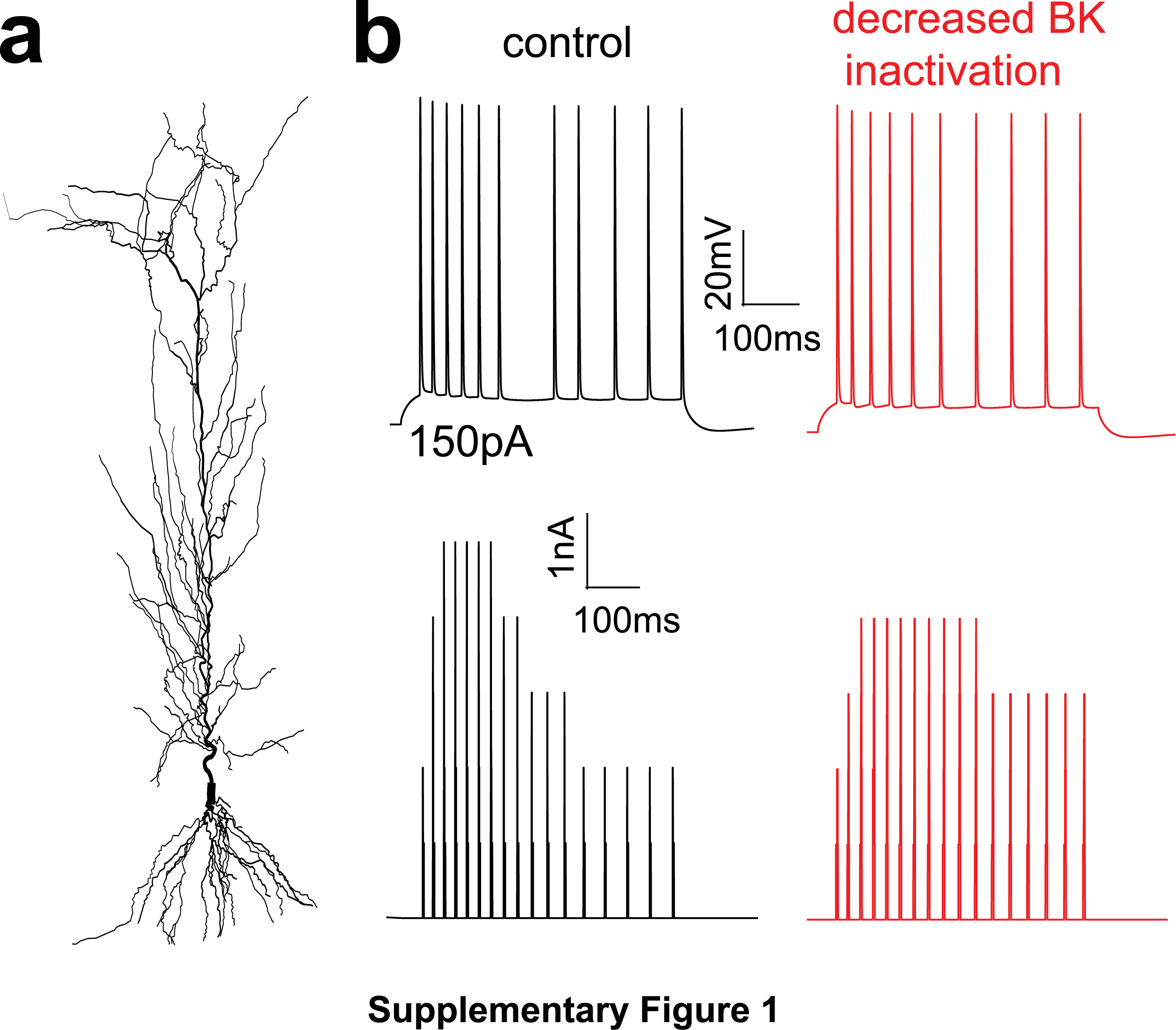
**

**Supplementary Figure 1. Simulating decreased BK current inactivation in a detailed CA1 neuron model**. (**a**) Schematic of the morphology of the CA1 neuron model used. (**b**) Train of action potentials (top) and recorded I_BK_ (bottom) in response to a 150 pA, 500 ms step-pulse stimulation at the soma under control conditions (black traces) and under conditions of decreased BK current inactivation.


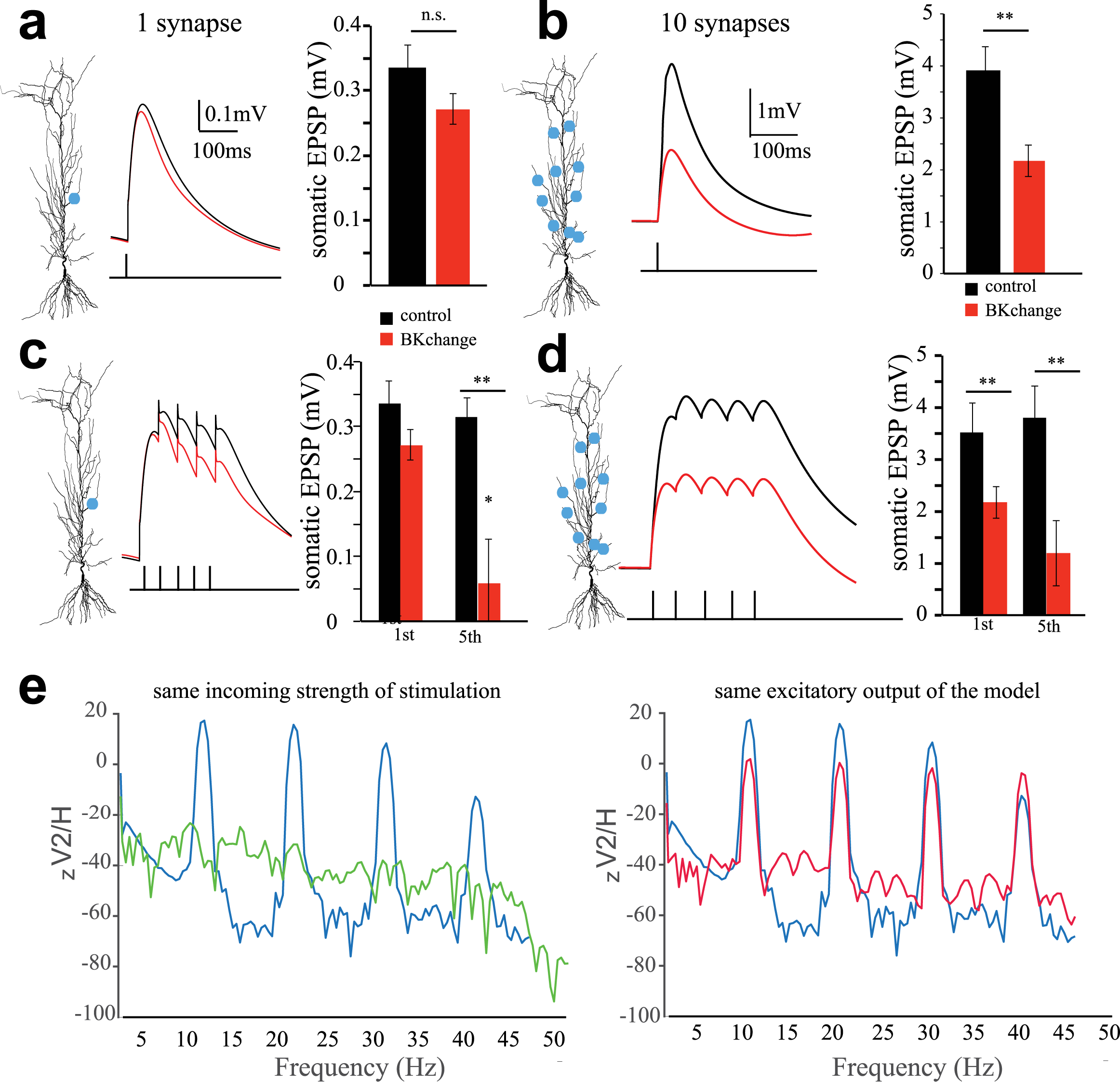


**Supplementary Figure 2.** **The effects of decreased BK current inactivation on synaptic integration and power spectra of cumulative synaptic activity**. Results are shown as representative traces and as bar graphs of the average of 10 simulation runs with each run activating a different dendrite or set of dendrites. (**a**) The somatic EPSP in response to a single synapse stimulation, showing that decreased BK current inactivation did not significantly affect the propagation of single synapse activation to the soma. (**b**) The somatic EPSP in response to stimulation of 10 synapses dispersed on different apical dendrites, showing that decreased BK current inactivation significantly decreased the somatic EPSP. (**c**) The somatic EPSP in response to 5-times 20 Hz stimulation of a single synapse, showing that decreased BK current inactivation significantly reduced the amplitude of the 5th somatic EPSP. (**d**) The somatic EPSP in response to 5-times 20 Hz stimulation of 10 dispersed synapses, showing that decreased BK current inactivation reduced the amplitude of the 1st and 5th somatic EPSP. (**e**) The CA1 neuron model was stimulated with 100 synapses dispersed at a theta frequency. The summed synaptic currents were analyzed for the power spectra. We found that the power of the theta frequency was significantly reduced when the stimulation parameters (i.e. the number of synapses stimulated) were the same between the two conditions (control and decreased BK current inactivation). If the number of synapses stimulated was increased in the ‘decreased BK current inactivation’ condition so that the output of the model neuron was similar to the control, then the power of the theta rhythm was increased, although still reduced compared to control. * *P* < 0.05; ** *P* < 0.01.

**Supplementary Figure 3. Metabolic parameters changed due to methyl donor-rich diet in F0 and F1**. (**a**) Metabolites in F0 father liver homogenates from control (CD) or methyl diet groups (MD); *n* = 5 mice per group. (**b**) Metabolites in liver homogenates from F1 offspring of CD and MD fathers. *n* = 5 mice per group. (**c**) F1 plasma lactate and urea levels. CD F1, *n* = 22 mice; MD F1, *n* = 23 mice. (**d**) Respiratory exchange level (RER) over 23 hours as a measure of relative lipid or carbohydrate metabolism. *n* = 16 mice per group. (**e**) Elevated focal steatosis in MD F1 offspring in H&E stained liver sections. (**a**) – (**d**) Data were compared across groups by unpaired t-tests. *P*-values are provided in the figure. * P < 0.05; ** *P* < 0.01; *** *P* < 0.001.

**Supplementary Tables**

**Supplementary Table 1. Behavioral analyses of MD F1 and CD F1 mice.** TwA: Two-way ANOVA; pd: effect of paternal diet; t: effect of trial; i: interaction; d: effect of day; si: effect of sound intensity.

|  | CD F1 offspring | | MD F1 offspring | | Statistical analysis |
| --- | --- | --- | --- | --- | --- |
|  | *n* | Mean +/- SEM | *n* | Mean +/- SEM |  |
| (a) Hidden version of the Morris water maze  Training trials:  - Escape latency (s)  Day 1  Day 2  Day 3  Day 4  Day 5  Day 6  Day 7  - Thigmotaxis (% time)  Day 1  Day 2  Day 3  Day 4  Day 5  Day 6  Day 7  - Distance travelled (cm)  Day 1  Day 2  Day 3  Day 4  Day 5  Day 6  Day 7  Probe trial:  - Distance traveled (cm)  - Cumulative proximity to target (cm)  - Thigmotaxis (% time)  - Swim speed (cm/s) | 20 | 44.940 +/- 2.801  40.539 +/- 2.827  29.664 +/- 3.465  27.091 +/- 2.984  19.007 +/- 2.842  14.271 +/- 2.701  18.622 +/- 3.548  58.18 +/- 3.268  34.88 +/- 3.107  21.30 +/- 2.791  15.79 +/- 3.133  9.447 +/- 2.836  5.030 +/- 1.305  3.414 +/- 1.175  891.3 +/- 58.65  896.7 +/- 60.89  660.6 +/- 78.91  615.8 +/- 68.36  416.4 +/- 69.53  301.0 +/- 59.74  396.2 +/- 79.50  1378 +/- 33.67  2563 +/- 87.07  6.974 +/- 1.929  23.00 +/- 0.5613 | 20 | 40.135 +/- 3.263  29.667 +/- 4.150  35.245 +/- 4.589  22.927 +/- 3.857  23.866 +/- 3.062  16.048 +/- 2.963  28.963 +/- 3.583  55.80 +/- 4.352  28.32 +/- 3.851  23.12 +/- 3.439  14.98 +/- 2.594  11.78 +/- 2.585  5.167 +/- 1.273  8.514 +/- 2.449  832.8 +/- 70.57  665.3 +/- 93.86  785.9 +/- 102.8  510.0 +/- 84.71  528.5 +/- 75.01  337.9 +/- 67.13  627.8 +/- 73.37  1385 +/- 43.61  2800 +/- 92.86  11.75 +/- 2.802  23.11 +/- 0.7277 | TwA:  pd, *P*=0.8605;  d, *P*<0.0001;  i, *P*=0.0202  TwA:  pd, *P*=0.9826;  d, *P*<0.0001;  i, *P*=0.3511  TwA:  pd, *P*=0.7507;  d, *P*<0.0001;  i, *P*=0.0299  t-test, *P*=0.9034  t-test, *P*=0.0713  t-test, *P*=0.1685  t-test, *P*=0.9001 |
| (b) Visible version of the Morris water maze  Escape latency (s)  Trial 1  Trial 2  Trial 3  Trial 4 | 20 | 16.285 +/- 2.265  17.135 +/- 2.439  9.797 +/- 1.886  10.995 +/- 1.464 | 20 | 16.025 +/- 2.881  17.320 +/- 2.306  9.325 +/- 1.927  10.037 +/- 1.084 | TwA:  pd, *P*=0.8220;  t, *P*=0.0001;  i, *P*=0.9937 |
| (c) Open field  - Distance travelled (cm)  - Number of rearings  - Total time spent in the center (s) | 25 | 13882 +/- 443.5  80.96 +/- 4.304  104.7 +/- 11.56 | 19 | 13295 +/- 516.4  83.37 +/- 5.077  90.67 +/- 13.16 | t-test, *P*=0.3923  t-test, *P*=0.7182  t-test, *P*=0.4296 |
| (d) Grip strength  Mean force (g)  2-paw  4-paw | 25 | 113.3 +/- 2.072  212.7 +/- 4.640 | 19 | 115.7 +/- 3.041  214.5 +/- 4.284 | t-test, *P*=0.5006  t-test, *P*=0.7827 |
| (e) Accelerating rotarod  Latency to fall (s)  Trial 1  Trial 2  Trial 3 | 25 | 62.48 +/- 8.234  94.04 +/- 10.47  108.0 +/- 10.44 | 19 | 68.00 +/- 12.06  90.79 +/- 12.63  113.7 +/- 12.40 | TwA:  pd, *P*=0.8406;  t, *P*<0.0001;  i, *P*=0.7859 |
| (f) Hot plate  Response latency (s)  First response  Second response | 24 | 13.07 +/- 0.5508  16.85 +/- 0.5844 | 19 | 12.58 +/- 0.5969  17.41 +/- 0.5831 | t-test, *P*=0.5532  t-test, *P*=0.5038 |
| (g) Acoustic startle reactivity  NB  70 dB  80 dB  85 dB  90 dB  100 dB  110 dB  120 dB | 25 | 72.53 +/- 10.79  144.6 +/- 15.27  231.9 +/- 21.99  509.5 +/- 42.12  1026 +/- 72.62  1824 +/- 77.66  2058 +/- 91.98  1993 +/- 95.19 | 19 | 75.41 +/- 13.98  150.6 +/- 16.43  271.7 +/- 36.88  596.5 +/- 65.62  1188 +/- 73.65  1938 +/- 94.33  2016 +/- 69.80  1994 +/- 89.80 | TwA:  pd, *P*=0.4473;  si, *P*<0.0001;  i, *P*=0.4401 |
| (h) Prepulse inhibition (%)  67 dB  69 dB  73 dB  81 dB  Global | 25 | 38.32 +/- 1.998  41.20 +/- 2.914  51.43 +/- 3.047  62.58 +/- 2.123  48.38 +/- 2.217 | 19 | 27.87 +/- 3.698  33.13 +/- 3.685  41.58 +/- 4.118  61.61 +/- 1.961  41.06 +/- 2.999 | TwA:  pd, *P*=0.0506;  si, *P*<0.0001;  i, *P*=0.0259  t-test, *P*=0.0510 |

**Supplementary Table 2.**  **Summary of hippocampal EEG recordings and awake EEG parameters and results for control and methyl diet F1 offspring.**

|  | CD F1 offspring | | MD F1 offspring | | Statistical analysis |
| --- | --- | --- | --- | --- | --- |
|  | *n* | Mean +/- SEM | *n* | Mean +/- SEM |  |
| Theta duration  (min/h)  - During motor activity  - During absence of motor activity  Motor activity | 11 | 5.548 +/- 0.5692  15.52 +/- 2.146  10.45 +/- 0.7972 | 11 | 3.236 +/- 0.9312  8.865 +/- 2.346  11.59 +/- 0.8720 | t-test, *P* = 0.0469  t-test, *P* = 0.0492  t-test, *P* = 0.3453 |

**Supplementary Table 3. Gene expression changes in hippocampus of MD F1 mice**. The table shows differentially expressed genes (FDR < 0.1) identified in a microarray-based comparison of hippocampal gene expression in CD F1 and MD F1 mice (per group, we used *n* = 6 pools of n = 4 mice each). Listed are probe IDs, gene symbols and descriptions, log fold changes (MD F1 / CD F1), *P* values and false discovery rates (FDR).

| Probe | Symbol | Description | logFC | *P* value | FDR |
| --- | --- | --- | --- | --- | --- |
| A_55_P2334927 |  |  | -0.46 | 2.91E-06 | 5.80% |
| A_55_P2252891 |  |  | -0.52 | 4.35E-06 | 5.80% |
| A_55_P2333126 |  |  | -0.59 | 5.53E-06 | 5.80% |
| A_55_P2007761 | Kcnmb2 | potassium large conductance calcium-activated channel, subfamily M, beta member 2 | -0.81 | 7.17E-06 | 5.80% |
| A_66_P109802 | EG13909 | predicted gene, EG13909 | -0.89 | 1.16E-05 | 7.52% |
| A_55_P2094044 | AU042671 | expressed sequence AU042671 | -0.43 | 2.10E-05 | 9.45% |
| A_51_P511511 | Stk33 | serine/threonine kinase 33 | 0.42 | 2.21E-05 | 9.45% |
| A_55_P2378129 |  |  | -0.66 | 3.55E-05 | 9.45% |
| A_55_P1955497 | LOC100048657 | hypothetical protein LOC100048657 | -0.82 | 3.60E-05 | 9.45% |
| A_55_P2157507 |  |  | -0.93 | 4.41E-05 | 9.45% |
| A_51_P196207 | Capsl | calcyphosine-like | 0.52 | 4.49E-05 | 9.45% |
| A_55_P1964533 | Lrrc43 | leucine rich repeat containing 43 | 0.45 | 4.55E-05 | 9.45% |
| A_55_P1981909 | Ptprt | protein tyrosine phosphatase, receptor type, T | -0.52 | 4.68E-05 | 9.45% |
| A_55_P2168490 |  |  | -1.03 | 5.13E-05 | 9.45% |
| A_55_P2007165 |  |  | -0.6 | 5.24E-05 | 9.45% |
| A_55_P2032362 |  |  | -0.76 | 5.62E-05 | 9.45% |
| A_55_P2081945 | 380650 | predicted gene, 380650 | -0.5 | 6.39E-05 | 9.45% |
| A_55_P2167898 | Nat9 | N-acetyltransferase 9 (GCN5-related, putative) | -0.46 | 6.75E-05 | 9.45% |
| A_52_P352735 | Ccdc153 | coiled-coil domain containing 153 | 0.49 | 6.78E-05 | 9.45% |
| A_55_P2413722 |  |  | -0.66 | 7.45E-05 | 9.45% |
| A_55_P2209273 |  |  | -1.28 | 7.79E-05 | 9.45% |
| A_55_P2035519 | Kif9 | kinesin family member 9 | 0.5 | 8.14E-05 | 9.45% |
| A_52_P44030 | Exoc3 | exocyst complex component 3 | -0.55 | 8.26E-05 | 9.45% |
| A_55_P2047809 | Snhg11 | small nucleolar RNA host gene 11 (non-protein coding) | -0.63 | 8.30E-05 | 9.45% |
| A_52_P287692 | Stk32c | serine/threonine kinase 32C | -0.51 | 8.63E-05 | 9.45% |
| A_51_P500344 | Mat2a | methionine adenosyltransferase II, alpha | -0.41 | 8.77E-05 | 9.45% |
| A_55_P2069765 | Kiss1 | KiSS-1 metastasis-suppressor | 0.59 | 8.92E-05 | 9.45% |
| A_55_P2100705 |  |  | -0.5 | 1.04E-04 | 9.51% |
| A_55_P1970655 |  |  | -0.45 | 1.05E-04 | 9.51% |
| A_51_P125986 | Gan | giant axonal neuropathy | -0.69 | 1.06E-04 | 9.51% |
| A_52_P503614 | Kcnj2 | potassium inwardly-rectifying channel, subfamily J, member 2 | -0.59 | 1.12E-04 | 9.77% |
| A_52_P572197 | Pank3 | pantothenate kinase 3 | -0.88 | 1.21E-04 | 9.79% |
| A_51_P193686 | 1700012B09 | RIKEN cDNA 1700012B09 gene | 0.59 | 1.24E-04 | 9.79% |

**Supplementary Table 4. Quantitative RT-PCR analyses of *Mat2a* and *Kcnmb2* expression in hippocampi from control (CD) and methyl diet (MD) F1 offspring.** *Actb* was used as internal control in the experiments shown. Note: qPCR experiments using additional housekeeping genes (*B2m*, *Gapdh* and *Gusb*) yielded similar results (data not shown).

|  | CD F1 offspring | | MD F1 offspring | | Statistical analysis |
| --- | --- | --- | --- | --- | --- |
|  | *n* | Mean +/- SEM | *n* | Mean +/- SEM |  |
| qPCR  Normalized expression  *Mat2a*  *Kcnmb2* | 4  9 | 1.00 +/- 0.0094  1.00 +/- 0.0443 | 5  6 | 0.7563 +/- 0.0234  0.8242 +/- 0.0747 | t-test, *P* < 0.0001  t-test, *P* = 0.0497 |

**Supplementary Table 5. Summary of Morris water maze data of control diet (CD) and methyl diet (MD) F1 offspring treated with either hippocampal control AAV or hippocampal Kcnmb2-overexpressing AAV.** ThrwA: Three-way ANOVA; TwA: Two-way ANOVA; pd: effect of paternal diet; d: effect of day; aav: effect of AAV treatment; i: paternal diet x AAV treatment interaction; i(d x pd): day x paternal diet interaction; i(d x aav): day x AAV treatment interaction.

|  | CD F1 control-AAV  (*n*=12 mice) | MD F1 control-AAV  (*n*=12 mice) | CD F1 Kcnmb2-AAV  (*n* = 8 mice) | MD F1 Kcnmb2-AAV  (*n* = 8 mice) | Statistical analysis |
| --- | --- | --- | --- | --- | --- |
|  | Mean +/- SEM | Mean +/- SEM | Mean +/- SEM | Mean +/- SEM |  |
| Hidden version of the Morris water maze  Training trials:  - Escape latencies (s)  Day 1  Day 2  Day 3  Day 4  Day 5  Day 6  Day 7  Probe trial:  - Swim speed during probe trial (cm/s)  - Thigmotaxis during probe trial (% time)  - Cumulative proximity to target during probe trial (cm) | 45.8 +/- 3.9  34.3 +/- 1.8  27.3 +/- 1.8  14.5 +/- 2.1  11.3 +/- 1.5  13.8+/- 2.8  10.8 +/- 1.9  15.8 +/- 0.5  8.2 +/- 1.7  1295 +/- 68.17 | 46.6 +/- 5.8  35.4 +/- 3.8  30.1 +/- 3.4  15.5 +/- 2.3  12.8 +/- 2.1  12.5 +/- 1.6  9.2 +/- 1.7  16.3 +/- 0.6  7.5 +/- 1.6  1495 +/- 74.69 | 45.6 +/- 5.9  32.6 +/- 4.9  30.1 +/- 5.9  12.4 +/- 2.7  10.4 +/- 1.2  11.9 +/- 4.0  6.9 +/- 2.1  16.5 +/- 0.6  7.6 +/- 2.9  1297 +/- 78.93 | 45.5 +/- 6.3  35.3 +/- 4.3  27.8 +/- 5.8  14.1 +/- 1.3  12.6 +/- 1.6  12.6 +/- 3.9  8.9 +/- 2.4  16.0 +/- 0.9  7.6 +/- 1.9  1365 +/-92.44 | ThrwA:  pd, *P*=0.6131  aav, *P*=0.5511  d, *P*<0.0001  i(d x pd), *P*=0.9987  i(d x aav), *P*=0.9992  TwA:  pd, *P*=0.9896  aav, *P*=0.8139  i, *P*=0.4327  TwA:  pd, *P*=0.8862  aav, *P*=0.8974  i, *P*=0.8682  TwA:  pd, *P*=0.0994  aav, *P*=0.4265  i, *P*=0.4136 |

**Supplementary Table 6. Summary of Morris water maze data of control diet (CD) and methyl diet (MD) F2 offspring.** TwA: Two-way ANOVA; gpd: effect of grandpaternal diet; d: effect of day; i: interaction.

|  | CD F2 offspring | | MD F2 offspring | | Statistical analysis |
| --- | --- | --- | --- | --- | --- |
|  | *n* | Mean +/- SEM | *n* | Mean +/- SEM |  |
| Hidden version of the Morris water maze  Escape latencies (s)  Day 1  Day 2  Day 3  Day 4  Day 5  Day 6  Day 7  Probe trial:  - Cumulative proximity to target (cm)  - Thigmotaxis (% time)  - Swim speed (cm/s) | 18 | 51.034 +/- 3.336  47.871 +/- 2.679  37.231 +/- 3.941  32.213 +/- 3.700  33.033 +/- 4.383  40.346 +/- 4.082  26.330 +/- 3.504  1490 +/- 115.20  15.35 +/- 2.714  24.95 +/- 1.944 | 16 | 49.860 +/- 2.879  45.090 +/- 3.988  36.433 +/- 4.428  29.456 +/- 3.320  31.268 +/- 4.244  32.555 +/- 4.539  24.674 +/- 4.005  1316 +/- 95.09  18.64 +/- 3.485  22.02 +/- 1.596 | TwA:  gpd, *P*=0.3408  d, *P*<0.0001  i, *P*=0.9688  t-test, *P*=0.2588  t-test, *P*=0.4564  t-test, *P*=0.2597 |

**Supplementary Table 7. Primers used for targeted DNA methylation (pyrosequencing) assays.**

| Type of primer | Name of primer | Primer sequence (5’-3’) |
| --- | --- | --- |
| Forward  Reverse  Sequencing  Sequencing  Sequencing  Sequencing  Sequencing | KCNMB2_P1_F1  KCNMB2_P2_F2  KCNMB2_P1_R1  KCNMB2_P2_R2  KCNMB2_P1_S1  KCNMB2_P1_S2  KCNMB2_P1_S3  KCNMB2_P2_S4  KCBMB2_P2_S5 | aggatttggaaatttaatttag  gtttggataggtttttatagagt  ctccctaactcataactttact  tcctaccttcaaaaattatc  ttagtaggtagggtggt  ggaagaaatgttgga  ttttaaaaatagtttgg  tttttaaatttttgtagatat  ggaaaggtaaagatga |

**Supplementary Table 8. Assay information for targeted DNA methylation (pyrosequencing) analyses.**

| Assay | Sequencing primer | Sequence to analyze (5’-3’) |
| --- | --- | --- |
| CpG 1 to 3  CpG 4 to 6  CpG 7 to 9  CpG 10 to 11  CpG 12 to 14 | KCNMB2_P1_S1  KCNMB2_P1_S2  KCNMB2_P1_S3  KCNMB2_P2_S4  KCNMB2_P2_S5 | YGTAGAGTTAGGAGGGAAGTTAGATTA  GGTTTYGTTTTTTTTTTAGTATGAAAAT  TYG  TTTATTATAGYGATGTTAATAGATAGTT  TATTATAGTATTTAATAAAAATAGAAGT  AGYGTGTTTGTTATAGGTTAATAAATTT  TATTTTTTGTAYG  ATYGGTTTTTATAGAGTTTYGGAGAAT  TAATTTTAAGTATGGTTGTTTAGYG  TTTGAGYGAGGAGTAAAGTTATGAGTT  AGYG  GGATYGGTGGTTGTGTGGAGGATTAT  AYGGGATTATTGTTATTAAAAATTAAGY  G |
